# Selecting optimal partitioning schemes for phylogenomic datasets

**DOI:** 10.1186/1471-2148-14-82

**Published:** 2014-04-17

**Authors:** Robert Lanfear, Brett Calcott, David Kainer, Christoph Mayer, Alexandros Stamatakis

**Affiliations:** 1 Ecology Evolution and Genetics, Research School of Biology, Australian National University, Canberra, ACT, Australia; 2 National Evolutionary Synthesis Center, Durham, NC, USA; 3 Philosophy Program, Research School of Social Sciences, Australian National University, Canberra, ACT, Australia; 4 Zoologisches Forschungsmuseum Alexander Koenig, Bonn, Germany; 5 The Exelixis Lab, Scientific Computing Group, Heidelberg Institute for Theoretical Studies, Heidelberg, Germany; 6 Karlsruhe Institute of Technology, Institute for Theoretical Informatics, Postfach 6980, 76128 Karlsruhe, Germany

**Keywords:** Model selection, Partitioning, Partitionfinder, BIC, AICc, AIC, Phylogenetics, Phylogenomics, Clustering, Hierarchical clustering

## Abstract

**Background:**

Partitioning involves estimating independent models of molecular evolution for different subsets of sites in a sequence alignment, and has been shown to improve phylogenetic inference. Current methods for estimating best-fit partitioning schemes, however, are only computationally feasible with datasets of fewer than 100 loci. This is a problem because datasets with thousands of loci are increasingly common in phylogenetics.

**Methods:**

We develop two novel methods for estimating best-fit partitioning schemes on large phylogenomic datasets: strict and relaxed hierarchical clustering. These methods use information from the underlying data to cluster together similar subsets of sites in an alignment, and build on clustering approaches that have been proposed elsewhere.

**Results:**

We compare the performance of our methods to each other, and to existing methods for selecting partitioning schemes. We demonstrate that while strict hierarchical clustering has the best computational efficiency on very large datasets, relaxed hierarchical clustering provides scalable efficiency and returns dramatically better partitioning schemes as assessed by common criteria such as AICc and BIC scores.

**Conclusions:**

These two methods provide the best current approaches to inferring partitioning schemes for very large datasets. We provide free open-source implementations of the methods in the PartitionFinder software. We hope that the use of these methods will help to improve the inferences made from large phylogenomic datasets.

## Background

Choosing an appropriate model of molecular evolution (model selection) is an important part of phylogenetics, and can affect the accuracy of phylogenetic trees, divergence dates, and model parameters [1-11]. One of the most important aspects of model selection is to find a model that can account for variation in the substitution process among the sites of the alignment. This variation may include differences in rates of evolution, base frequencies, and substitution patterns, and the challenge is to account for all such variation found in any given dataset. There are many different ways to approach this problem, of which the simplest and most widely used is partitioning. In the broadest sense, partitioning involves estimating independent models of molecular evolution for different groups of sites in an alignment. These groups of sites are often user-defined (in which case we call them ‘data blocks’ here), for example based on genes and codon positions [7]. It is also increasingly common to refine user-defined partitioning scheme by combining similar data blocks algorithmically [2]. A vast number of phylogenetic studies have used partitioned models of molecular evolution, and it is widely appreciated that partitioning often leads to large improvements of the fit of the model to the data (see e.g. [2]). Many studies also report that partitioning has improved phylogenetic inference, including the estimation of tree topologies, branch lengths, and divergence dates [6,10,12-14].

Partitioning is one of many methods to account for variation in substitution processes among sites. Some approaches automatically assign sites to different substitution models (e.g. [15,16]), and others estimate more than one model of molecular evolution for each site (e.g. [17-19]). Many of these methods are better and more elegant than the form of partitioning we focus on here, because they do not rely on user-defined data blocks and can more effectively scale to the true variation in substitution processes present in the data. However, partitioning remains the most widely-used method to account for variation in rates and patterns of substitution among sites [9,17,20,21]. Its enduring popularity is part historical contingency and part practical: many of the superior methods are more recent and have not yet become widely adopted, and partitioning is implemented in many popular phylogenetic inference programs. Most importantly for this study, partitioning is still the most practical method with which to account for variation in rates and patterns of substitution in very large datasets. Because of this, it is important that we work to ensure that partitioned models of molecular evolution are as accurate as possible, particularly when they are applied to large datasets, and that is the focus of this study.

It is important to note that all of the commonly used methods to account for variation in substitution patterns among sites (including partitioning) assume that sequences evolved under a stationary, reversible, and homogeneous process. These assumptions are necessary to make the methods efficient enough to allow for searches of phylogenetic tree space, although they are far from guaranteed to hold for empirical datasets (e.g. [22]). It is possible to relax these assumptions, but the computational cost of doing so is extremely high and precludes effective tree searches in all but the simplest cases. So for the time being it is necessary to make these assumptions in order to estimate tree topologies from very large datasets.

The biggest challenge in partitioning is to select the most appropriate partitioning scheme for a given alignment, i.e. to divide the alignment into groups of sites that account for variation in patterns of molecular evolution, while avoiding over- or under-parameterisation [2,4]. To select a partitioning scheme, phylogeneticists typically start by grouping together putatively similar sites in an alignment into homogenous data blocks, using *a priori* knowledge of the variation in patterns of molecular evolution among sites [7,10]. The challenge is then to find an optimal partitioning scheme by combining sufficiently similar data blocks, which is usually done by finding the combination of data blocks that minimises a metric such as the corrected Akaike’s Information Criterion (AICc) or the Bayesian Information Criterion (BIC) [2]. For smaller datasets of up to about 100 initial data blocks, this optimisation step can be achieved automatically using a greedy heuristic search algorithm implemented in the software PartitionFinder [2]. However, recent reductions in DNA sequencing costs mean that it is now routine to produce very large ‘phylogenomic’ datasets which can contain hundreds or thousands of loci [23-25]. Current methods [2] are not computationally efficient enough to optimise partitioning schemes for these datasets. For example, the greedy algorithm implemented in PartitionFinder would have to analyse almost 9 million subsets of sites to estimate the optimal partitioning scheme for a sequence alignment of 1000 protein-coding loci, which is well beyond the bounds of practicality. This is a problem, because we have no methods to optimise partitioning schemes for the largest, and potentially most useful, datasets in phylogenetics.

Hierarchical clustering is a statistical method that has some attractive properties for optimising partitioning schemes for phylogenomic datasets. To use hierarchical clustering to optimise partitioning schemes, molecular evolutionary parameters (such as base frequencies and rates of molecular evolution) are first estimated for each initial data block, and data blocks are then combined based on the similarity of their parameter estimates. Hierarchical clustering and related methods (such as *k*-means clustering) have been used to select partitioning schemes in a number of previous studies with datasets of various sizes [4,26-30]. Hierarchical clustering is far more computationally efficient than the greedy algorithm implemented in PartitionFinder: if N is the number of data blocks specified by the user, hierarchical clustering is *O(N)*, while the greedy algorithm is *O(N*^*2*^*)*. For example, with an alignment of 1000 protein-coding genes, the strict hierarchical clustering approach we describe below requires the analysis of only 1999 subsets of sites (see methods, below), which is more than 3 orders of magnitude more efficient than existing approaches.

One drawback of hierarchical clustering is that a-priori decisions have to be made about the best way to determine the ‘similarity’ of different data blocks. Researchers typically estimate up to four parameter categories for each data block: (i) a parameter to describe the overall substitution rate of that data block (often called a rate multiplier); (ii) one or more parameters to describe the relative rates at which nucleotides replace each other (e.g. the 6 parameters of the General Time Reversible (GTR) model, known as the rate matrix); (iii) parameters to describe the proportions of nucleotides or amino acids in the data block (base or amino acid frequencies); and (iv) one or two parameters to describe the distribution of substitution rates among sites (a proportion of invariant sites and/or an alpha parameter describing a gamma distribution). In principle, data block similarity can be defined using any combination of these parameters. However, different studies have used different parameter combinations, and there has been no attempt to systematically understand the best way to define the similarity of different data blocks when estimating partitioning schemes [4,26-30].

In this study, we set out to investigate the performance of hierarchical clustering approaches for optimising partitioning schemes for phylogenomic datasets. We first developed a generalised strict hierarchical clustering method that allows the user to define relative importance of different model parameters when defining the similarity of subsets. We found that the choice of weighting scheme can have very large effects on the performance of the algorithm, and that regardless of the weighting scheme strict hierarchical clustering always performed substantially worse than the existing greedy algorithm. To remedy this, we developed a new method, which we call relaxed hierarchical clustering, that incorporates many of the benefits of strict hierarchical clustering while avoiding many of its disadvantages. We show that relaxed hierarchical clustering outperforms strict hierarchical clustering on all of the datasets that we examined. The computational demands of this method can be scaled to the dataset and computational resources available. It is therefore a pragmatic approach to estimating best-fit partitioning schemes on phylogenomic datasets, where more rigorous methods are computationally infeasible.

We have implemented all of the methods described in this study in the open-source software PartitionFinder, which is available for download from http://www.robertlanfear.com/partitionfinder. The PartitionFinder source code is available from https://github.com/brettc/partitionfinder/.

## Methods

### Terminology

Following previous studies [2,4], we define a “data block” as a user-specified set of sites in an alignment. A data block may consist of a contiguous set of sites (e.g. an intron), or a non-contiguous set (e.g. 1^st^ codon positions of a protein coding gene). A “subset” is a collection of one or more data blocks. Therefore, all data blocks are also subsets, but the converse is not true. A “partitioning scheme” is a collection of subsets that includes all data blocks once and only once. We do not use the term “partition” because it has conflicting meanings in phylogenetics and set theory – in phylogenetics a “partition” is used colloquially to denote what we call a “subset” here, whereas in set theory it defines what we call a “partitioning scheme” [2].

### Strict hierarchical clustering algorithm

We developed a strict hierarchical clustering algorithm inspired by a popular previous implementation [4], with some improvements. This algorithm is extremely efficient – given a set of N initial data blocks it creates a set of N partitioning schemes with between 1 and N subsets, and then selects the best partitioning scheme from this set. The algorithm has seven steps, which we summarise here and describe in more detail below:

1. Estimate a phylogenetic tree topology from the sequence alignment;

2. Start with a partitioning scheme that has all user-defined data blocks assigned to independent subsets;

3. Calculate the ML model parameters and log likelihood of each subset in the current partitioning scheme;

4. Calculate the similarity of all pairs of subsets in the current partitioning scheme;

5. Create a new partitioning scheme by combining the two most similar subsets in the current partitioning scheme;

6. Return to step 3, until a partitioning scheme with all sites combined into a single subset is created (i.e. terminate after N iterations);

7. Choose the best-fit partitioning scheme based on information theoretic metrics.

In principle this algorithm could be applied to DNA or amino acid alignments, but for simplicity we focus only on DNA alignments in this study.

All ML calculations in this algorithm are performed with a modified version of RAxML [21] available at https://github.com/brettc/standard-RAxML because RAxML is the most widely-used and computationally efficient software for analysing extremely large alignments. We substantially modified the PartitionFinder code (https://github.com/brettc/partitionfinder) to enable it to perform model selection and partitioning scheme selection by calling RAxML, and parsing the output produced by RAxML.

In step 1 of the strict hierarchical clustering algorithm, we estimate a maximum parsimony (MP) starting topology in RAxML which is then fixed for the rest of the analysis. Fixing the topology is crucial in increasing computational efficiency when searching for best-fit partitioning schemes [2]. Although MP is known to perform poorly relative to maximum likelihood (ML) when estimating phylogenetic trees, previous studies have shown that any non-random tree topology is adequate for accurate model selection [31,32]. Nevertheless, our implementation of this algorithm in PartitionFinder allows users to specify a starting tree topology calculated using any method, so that datasets for which MP has known issues may still be analysed rigorously.

In step 2 we calculate the log likelihood and parameters of a GTR + G model on each new subset of sites using RAxML. A new subset of sites is defined as a subset that the algorithm has not yet encountered. The log likelihood and ML parameters of each subset are then stored in memory so that they do not have to be recalculated in subsequent iterations of the algorithm. We use the GTR + G model rather than the GTR + I + G model because the ‘I’ parameter, which describes the proportion of invariant sites, is not independent from the ‘G’ parameter, which describes the gamma distribution of rates across sites, making it impossible to estimate both parameters accurately [33]. This dependency between ‘G’ and ‘I’ compromises attempts to infer the similarity of subsets using their parameter estimates (step 3). In principle, however, step 2 could include the selection of the best model of molecular evolution for any given subset.

In step 3 we calculate the similarity of subsets based on their ML model parameters. To do this, we group parameters into four categories and use a distance metric that allows users to specify the relative importance of different parameter categories. The four parameter categories are: (i) the overall rate of evolution of the subset, calculated as the sum of the maximum likelihood branch lengths for that subset; (ii) the 6 parameters of the General Time Reversible (GTR) model; (iii) the four base frequencies; and (iv) the alpha parameter that describes the gamma distribution of rates across sites. The parameters from categories (ii) and (iii) are not independent of each other, but we include both because we do not have prior information on which parameters are more important, or which may be most useful for optimising partitioning schemes. To calculate the similarity of all pairs of subsets, we first calculate a pairwise Euclidean distance matrix for each of the four parameter categories. We then normalise each distance matrix so that the maximum distance is one, and then scale each matrix by a user-specified weight (set using the ‘--weights’ command line option in PartitionFinder v1.1.0). The similarity of a pair of subsets is then calculated as the sum of the distances across the four matrices, which gives the Manhattan or city block distance between a pair of subsets. In this approach, the user-specified weights have a natural interpretation as the relative importance of different parameters in defining subset similarity.

This approach to calculating subset similarity has a number of advantages over previous methods. Many previous approaches have used fewer than the four categories we define to calculate subset similarity, and most have implicitly assumed that all parameter categories are equally important in determining subset similarity [4]. In contrast, our method allows for any combination of parameter categories to be specified, and for the relative importance of each category to be specified. For example, a parameter category can be excluded from similarity estimates by setting its weight to zero. Similarly, a parameter category can be defined as tenfold less important than other categories by setting its weight to 0.1, and the weights of the other categories to 1. Another limitation of previous clustering approaches is that they have estimated the parameters of larger subsets directly from the parameter estimates of subsets they contain [4]. This approach is problematic because it is difficult to predict how the information in two smaller subsets will combine to determine the parameters of the larger subset, and simply averaging the ML parameters of the smaller subsets is unlikely to produce parameters close to the ML parameters for the larger subset. Furthermore, error in the parameter estimates of the smaller subsets may limit their accuracy in the first place [2]. Our approach circumvents these problems by calculating ML parameter estimates for every subset that is analysed, including subsets that were created by merging together two smaller subsets. This approach ensures that the hierarchical clustering procedure is as accurate as possible, given the limitations of estimating model parameters from finite datasets.

In each iteration of our algorithm, we find the most similar pair of subsets from the focal partitioning scheme (step 4), and then merge these subsets to create a new subset and a new partitioning scheme (step 5). In this manner, the algorithm iteratively merges subsets to create a set of N partitioning schemes from N initial data blocks. These N schemes contain from 1 to N subsets. The final step of the algorithm (step 7) simply involves comparing the information theoretic score (e.g. AIC, AICc, or BIC) of all N partitioning schemes, and choosing the scheme with the best score. Choosing the best partitioning scheme does not involve any further ML calculations, because the log likelihood of each partitioning scheme can be calculated from the sum of the log likelihoods of the subsets contained in that scheme [2].

### Relaxed hierarchical clustering algorithm

The strict hierarchical clustering algorithm is computationally efficient, but it has some obvious drawbacks. First, it can merge subsets that make the information theoretic score of a partitioning scheme worse, rather than better. This is because there is no guarantee that any given measure of ‘similarity’ will translate into an improvement in the information theoretic score. Second, even if a given similarity measure does translate into robust improvements in the information theoretic score, the algorithm may be misled when the accuracy of ML parameter estimates is limited, as can be the case with small subsets [2].

To overcome these limitations, we propose a relaxed hierarchical clustering algorithm. This algorithm has eight steps:

1-4. Identical to strict hierarchical clustering

5. Select the top P% of most similar subset pairs;

6. Create S new partitioning schemes, each of which includes one of the subset pairs from step 5;

7. Choose the partitioning scheme from step 6 with the best information-theoretic score (AIC, AICc, BIC);

8. Return to step 3, until no further improvements in the information theoretic score are found;

Steps 1–4 proceed precisely as in the strict hierarchical clustering algorithm. In step 5 we create a ranked list of all possible subset pairs from the current partitioning scheme, where the rank is defined by the similarity of the subsets. We then use a user-defined percentage, P (‘--rcluster-percent’ in PartitionFinder), to choose the S most similar subsets pairs. In step 6 we create a new partitioning scheme for each of the S subset pairs, by merging the two subsets in each pair and calculating the new log-likelihood and maximum likelihood parameter estimates. In step 7, we calculate the information theoretic score (AIC, AICc, BIC) of each of the S new partitioning schemes, and select the partitioning scheme with the best score. The algorithm then iterates (step 8) until no further improvements in the information theoretic score can be found.

The key difference between the relaxed and strict hierarchical clustering algorithms is the ability to set the parameter P, which controls the thoroughness of the heuristic search algorithm. When P is set to 0%, the relaxed clustering algorithm will behave similarly to the strict hierarchical clustering algorithm, and only evaluate the single partitioning scheme that includes the most similar pair of subsets (although it differs insofar as the relaxed clustering algorithm is a hill-climbing algorithm, while the strict hierarchical clustering algorithm is not). When P is set to 100%, the relaxed clustering algorithm will behave similarly to the existing greedy algorithm in PartitionFinder [2], and evaluate all possible subset pairs at each iteration of the algorithm. Larger values of P will take more computational time, but are also likely to produce better solutions because they will search the space of partitioning schemes more thoroughly. In preliminary analyses we observed that even very small values of P (e.g. 0.1-1.0 percent) can often lead to the discovery of partitioning schemes that dramatically outperform those found by the strict hierarchical clustering algorithm.

### Datasets

As described above, we expect both of the new methods we describe here to perform worse than the greedy algorithm implemented in PartitionFinder, simply because they are less thorough heuristic searches. The true utility of the new methods is to find partitioning schemes for datasets that are too large to analyse with existing methods [2]. Nevertheless, to properly assess the new algorithms described here, it is necessary to compare them to existing approaches. Because of this, we focussed our analyses on data sets to which we could apply both the new and existing methods.

We used 10 publicly available datasets (Table 1) to compare the clustering methods to existing approaches. These datasets comprise a range of different sequence types (exons, introns, rRNAs, mithochondrial DNA, nuclear DNA), and come from a range of different taxa. The largest dataset comes from a phylogenomic study of birds (Hackett_2008, Table 1), and comprises 171 taxa, 52383 sites, and 168 data blocks. This dataset is close to the upper size limit of datasets that can be analysed using the greedy algorithm implemented in PartitionFinder 1.1.0 [2], so represents the practical limit of datasets that we can include in this study. In two cases (the Fong_2012 and Pyron_2011 datasets, Table 1) we reduced the number of taxa in the original dataset, by removing the taxa with the most gaps, in order that we could analyze the dataset using the greedy algorithm in PartitionFinder. Precise details of the taxa we removed are provided in the figShare repository associated with this article (http://dx.doi.org/10.6084/m9.figshare.938920). Removing taxa does not reduce the complexity of the task of selecting partitioning schemes, but simply reduces the computational burden of analysing each subset. Note that this is done to provide a suitable set of test datasets for comparing new and old methods, and we do not mean to imply that partitioning schemes estimated from reduced-taxon datasets should be used on the full-taxon dataset. All of the datasets, as well as the associated input files for PartitionFinder, are available from figShare (http://dx.doi.org/10.6084/m9.figshare.938920), and references for the datasets and the studies that they are associated with are provided in Table 1.

**Table 1:** Details of the 10 datasets used in this study

**Dataset name**	**Clade (common)**	**Clade (Latin)**	**Taxa**	**Sites**	**Data blocks**	**Study reference**	**Dataset reference**
Ward_2010	Ants	Dolichoderinae	54	9173	27	[34]	NA
Wainwright_2012	Fishes	Acanthomorpha	188	8439	30	[35]	[36]
Pyron_2011	Amphibians	Amphibia	18	12712	34	[37]	[38]
Li_2008	Fishes	Actinopterygii	56	7995	30	[4]	NA
Leavitt_2013	Grasshoppers	Acridoidea	34	15404	87	[12]	NA
Kaffenberger_2011	Frogs	Gephyromantis	54	6548	26	[39]	[40]
Irisarri_2012	Frogs	Neobatrachia	37	11136	34	[41]	[42]
Hackett_2008	Birds	Aves	171	52383	168	[43]	NA
Fong_2012	Vertebrates	Vertebrata	16	25919	168	[44]	[45]
Endicott_2008	Humans	Homo sapiens	179	13857	41	[46]	NA

The original study describing each dataset is referenced, the dataset itself is also referenced where it is archived under a separate DOI.

### Analyses

We exhaustively compared the two new algorithms to existing methods using the largest dataset in this study (Hackett_2008, Table 1). Based on the results of these analyses, we compared the two new algorithms to existing methods across the ten datasets described in Table 1. The analyses were run in PartitionFinder version 1.1.0 with the following settings common to all analyses: we used the RAxML version of PartitionFinder developed for this study (i.e. using the ‘--raxml’ commandline option, see above), because the older PhyML version of PartitionFinder is not computationally efficient enough to analyse the very large datasets that are the focus of this study (see above); all analyses were performed twice – once with model selection performed under the AICc, and once under the BIC; all branch lengths were set to ‘linked’ in all analyses, meaning that relative branch lengths were estimated at the start of the analysis using a GTR + G model in RAxML, and that these relative branch lengths were then fixed for the rest of the analysis, with each subset afforded its own rate multiplier to account for differences in rates of evolution between subsets [2]; only the GTR + G model of evolution was considered (see above). We do not consider analyses using the AIC, because the AICc should be preferred to the AIC in all cases [47].

We note that the approach we have implemented here, using a rate multiplier and a single set of molecular branch lengths, does not allow for heterotachy (variation in the pattern of rates among sites over time), although this is known to be an important source of variation in patterns of substitution [1,11]. In principle, our approach can account for heterotachy by allowing each subset to have an independent set of branch lengths, and this can be achieved in PartitionFinder by setting ‘branchlengths’ option to ‘unlinked’. However, in practice this way of accounting for heterotachy adds so many parameters to the overall model that it is inferior to using a rate multiplier. A better approach is to use a covarion model or a mixture of branch lengths [1,11], but since our focus here is producing partitioning schemes for very large datasets that can be subsequently analysed in RAxML, and since neither of these models is available in RAxML, we do not consider them further here.

For every analysis, we recorded: (i) the best partitioning scheme and it’s information theoretic score (i.e. AICc or BIC score); (ii) the information theoretic score of each partitioning scheme visited by each algorithm during the heuristic search; and (iii) the time taken to complete the analysis on a desktop a Mac Pro with 2 2.26 GHz Quad-Core Intel Xeon processors and 32GB RAM. The details of the absolute computational times are not important, but a comparison of the analysis times is informative (see below) because it allows us to empirically compare the computational efficiency of the different methods.

#### *Analyses using the phylogenomic bird dataset*

For the phylogenomic dataset from birds we first removed all sites in the alignment that were removed by the original authors [43], and then defined data blocks based on each intron, and each codon position in each exon. This resulted in a total of 168 data blocks. We then performed a total of 12,002 searches for partitioning schemes on this dataset, described below.

We performed 2 searches for optimal partitioning schemes using the greedy algorithm [2]: one with the AICc, and one with the BIC.

We performed 2000 searches for optimal partitioning schemes using the strict hierarchical clustering algorithm described above. The 2000 searches comprise 1000 searches using the BIC and 1000 using the AICc, where each search used one of 1000 distinct clustering weights (the ‘--weights’ commandline option in PartitionFinder). The clustering weights are defined by a vector of four numbers that specify the relative importance of four parameter categories (the overall subset rate, the base frequencies, the GTR model parameters, and the alpha parameter of the gamma distribution; see above). Analysing 1000 sets of weights allows us to empirically compare the performance of different weighting schemes, and to determine the relative importance of the different parameter categories when searching for partitioning schemes, as well as the variation in the algorithm’s performance under different weighting schemes. The first 15 sets of weights comprise all possible combinations of setting at least one weight to 1.0, and other weights to 0.0 (setting all weights to 0.0 is nonsensical, as it would lead to all subsets appearing to be equally similar). These represent 15 of the 16 corners of a four dimensional hypercube, and allow us to compare the 15 cases where either all parameter categories are given equal weight (i.e. --weights “1, 1, 1, 1”) or where one or more parameters are given zero weight (e.g. --weights “1, 0, 0, 1”). The other 985 points were chosen using Latin Hypercube Sampling in the ‘lhs’ package, version 0.1 in R [48]. This procedure ensures that the sampled points are relatively evenly distributed in four-dimensional space, and is a more efficient way of sampling high-dimensional space than using a grid-based sampling scheme.

We performed 10,000 searches for optimal partitioning schemes using the relaxed clustering algorithm described above. These 10,000 searches comprised 5000 searches using the AICc, and 5000 using the BIC, each of which was performed with 1000 different clustering weights, and at 5 different values of the parameter P. The 1000 weighting schemes we used were identical to those used above, and the values of the parameter P (which defines the percentage of possible partitioning schemes that are considered at each step of the relaxed clustering algorithm) that we used were 1%, 2%, 5%, 10%, and 20%.

The results of all 12002 analyses presented here are available at figShare (http://dx.doi.org/10.6084/m9.figshare.938920).

#### *Analyses across all datasets*

Based on the results of our analyses of the phylogenomic bird dataset, we set some pragmatic default values for the clustering weights and the P parameter (see below). We then analysed the performance of the greedy algorithm, the strict hierarchical clustering algorithm, and the relaxed hierarchical clustering algorithm across all 10 datasets in Table 1 using these default settings. We compared both the computational time and the performance of all three algorithms across all 10 published datasets. This involved a total of 60 analyses: 10 datasets, 2 information theoretic scores (AICc, and BIC), and 3 algorithms (greedy, strict clustering, and relaxed clustering). Details of all of the datasets are given in Table 1, input files for PartitionFinder, and results of these analyses are available from figShare (http://dx.doi.org/10.6084/m9.figshare.938920).

## Results and discussion

All three algorithms we discuss in this paper start with a user-defined set of data blocks, and progressively merge data blocks to improve the information-theoretic score of the partitioning scheme. Better algorithms will lead to larger improvements in the information theoretic score. We discuss algorithm performance below in two ways: in terms of the amount (in AICc or BIC units) that they improve the score of the partitioning scheme relative to the starting scheme which has each data block assigned to an independent subset; and in terms of the percentage improvement that an algorithm achieves relative to the existing greedy algorithm in PartitionFinder. Thus, a good algorithm will score highly on both counts.

### Strict hierarchical clustering

**Figure 1:**
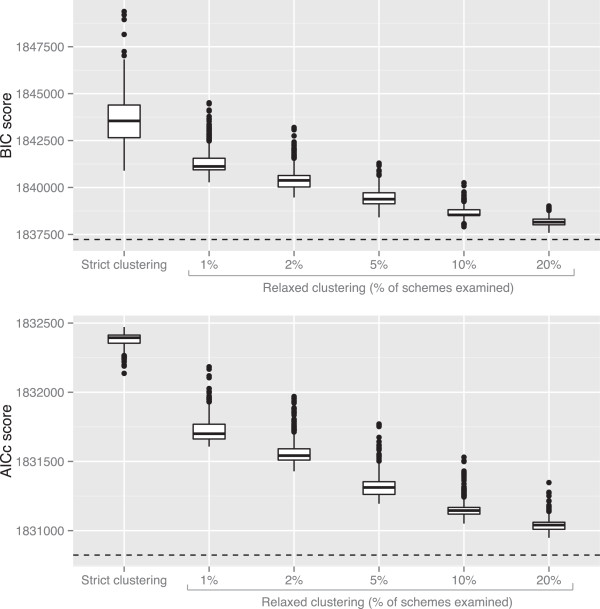
**The strict clustering algorithm performs poorly, but the relaxed clustering algorithm performs almost as well as the greedy algorithm.** All analyses were conducted on a phylogenomic dataset of birds (Table 1, Hackett_2008). Note that lower scores indicate a better fit of the model to the data. The dashed line in each plot shows the score of the best partitioning scheme found by the greedy algorithm. Each boxplot represent the distribution of scores for 1000 runs of the strict or relaxed clustering algorithms, where each run uses a different definition of the similarity of two subsets (see main text). The figure shows that the relaxed clustering algorithm’s performance approaches that of the greedy algorithm as P increases, and that analysing 10% of partitioning schemes results in information theoretic scores that are very close to that of the greedy algorithm.

The strict hierarchical clustering algorithm performed substantially worse than the greedy algorithm on the phylogenomic bird dataset (Figure 1, Table 1). This was the case regardless of the way in which subset similarity was defined, or whether partitioning schemes were selected using the AICc or the BIC (Figure 1). The greedy algorithm improved the AICc and BIC scores of the partitioning scheme by 1689 and 13013 units respectively. Across all 1000 different sets of clustering weights analysed, the best-scoring partitioning schemes found by the strict hierarchical clustering algorithm improved the AICc and BIC scores by 376 and 9347 units respectively (Figure 1). These improvements represent 22% and 72% of the potential improvement in AICc and BIC scores estimated from the greedy algorithm.

The performance of the strict hierarchical clustering algorithm also varied substantially depending on the way in which subset similarity was defined. Across all 1000 different sets of clustering weights analysed, the worst-scoring partitioning schemes found by the strict hierarchical clustering algorithm improved the AICc and BIC scores by 42 and 862 units respectively (Figure 1). These improvements represent 2% and 7% of the potential improvement in AICc and BIC scores estimated from the greedy algorithm. The mean improvement in AICc and BIC scores across all 1000 different sets of clustering weights was 8% and 51% of the potential improvement in AICc and BIC scores.

**Figure 2:**
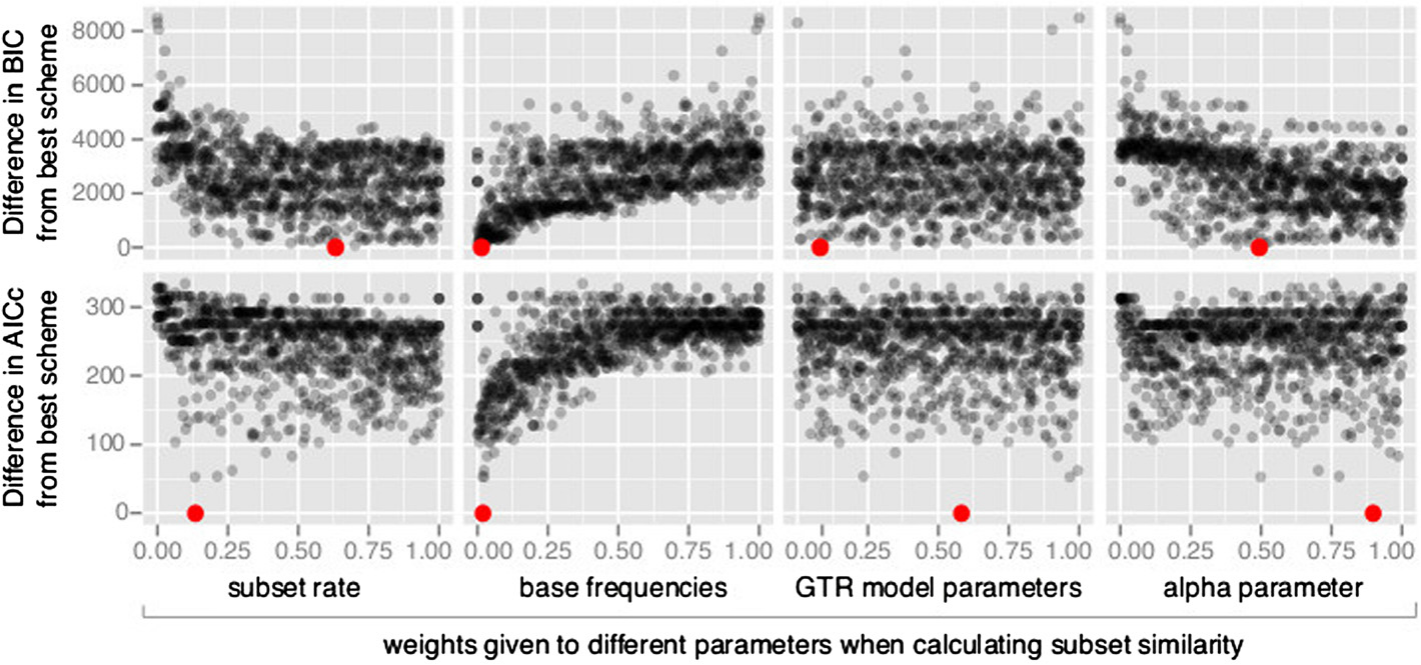
**The performance of the strict clustering algorithm varies dramatically depending on the weighting schemes used to define subset similarity.** The Y axis shows the difference in the AICc or BIC score compared to the best scheme found by the strict hierarchical clustering algorithm on the phylogenomic dataset from birds (Table 1). The X axes show the weights assigned to each of four parameter classes used to define subset similarity. Each panel shows 1000 data points, where each datapoint represents a single run of the strict hierarchical clustering algorithm under a particular weighting scheme. The set of four weights under which the best scheme was found by the strict hierarchical clustering algorithm are shown in red.

The weights used to define subset similarity have a complex relationship to the performance of the strict hierarchical clustering algorithm. Figure 2 shows that the performance of the strict hierarchical clustering algorithm was better when the weights given to the overall rate of a subset and the alpha parameter were higher, and when the weight given to the base frequencies of a subset was lower. However, all of these relationships show substantial variation. Furthermore, the set of weights that resulted in the best partitioning scheme (shown in red dots on Figure 2) differed depending on whether the AICc or the BIC was used to evaluate partitioning schemes, and would be very difficult to predict from first principles. One of the clearest results from this analysis is that grouping together subsets based on their base frequencies always led to worse performance for this dataset (Figure 2). This suggests that base frequencies can provide misleading information on subset similarity. This is likely to be most severe when subsets are small and base frequencies are estimated from limited data, which in turn will be most problematic at the start of the algorithm.

These results suggest that in most practical cases (in which many fewer than 1000 different definitions of subset similarity would be compared), the strict hierarchical clustering algorithm is likely to perform very poorly. Although some methods of defining subset similarity performed better than others, our results suggest that there is no one method of defining subset similarity that works well for the duration of the algorithm. This is likely to be because the parameters of molecular evolution that we are able to measure (overall rate of evolution, base frequencies, GTR model parameters, and alpha parameter) are not sufficient to determine whether clustering a given pair of subsets will result in an improvement of the AICc or BIC scores. As a result, the algorithm often clusters together subsets that result in a worsening of the AICc or BIC score. This problem is compounded by the fact that it is difficult to predict, either from first principles or empirical tests (Figure 2), the best way to define subset similarity given the parameters that we can measure.

### Performance of the relaxed hierarchical clustering algorithm

The relaxed hierarchical clustering algorithm performed better than the strict hierarchical clustering algorithm, and its performance approached that of the existing greedy algorithm (Figure 1). When 20% of all possible partitioning schemes were examined, the best-scoring partitioning schemes found by the relaxed hierarchical clustering algorithm improved the AICc and BIC scores by 1565 and 12655 units respectively (Figure 1). These improvements represent 93% and 97% of the potential improvement in AICc and BIC scores estimated from the greedy algorithm.

The performance of the relaxed hierarchical clustering algorithm improved as the percentage of schemes examined was increased (Figure 1). When 1% of all possible partitioning schemes were examined the mean improvement in AICc and BIC scores was 785 and 8903 units respectively. These improvements represent 46% and 68% of the potential improvement in AICc and BIC scores estimated from the greedy algorithm. These improvements increased with the percentage of all possible partitioning schemes that were examined, rising to >80% when 10% of schemes were examined, and >90% when 20% of schemes were examined. In Figure 1, this is demonstrated by the AICc and BIC scores from the relaxed clustering algorithm approaching those from the greedy algorithm as P increases. Concomitant with this improvement, dependence of the relaxed hierarchical clustering algorithm on the way in which subset similarity is defined decreased as the percentage of schemes examined increased (demonstrated by the reduction of the height of the boxes in Figure 1).

These results suggest that although our estimates of subset similarity are highly imperfect, they do contain information that can be used to help optimise partitioning schemes more efficiently. Unlike the strict hierarchical clustering algorithm, the relaxed hierarchical clustering algorithm does not rely solely on the estimated similarity of subsets in order to decide whether to cluster them together. Instead, it considers a collection of the most similar pairs of subsets and then chooses the pair that gives the largest improvement in the AICc or BIC score. This approach circumvents the limitation of the strict hierarchical clustering method by reducing the reliance of the algorithm on the estimates of subset similarity.

### The performance of the strict and relaxed clustering algorithms on 10 datasets

To ensure that the results we obtained on the phylogenomic dataset of birds were not idiosyncratic to a single dataset, we compared the strict and relaxed clustering algorithms to each other and to the greedy algorithm on a collection of 10 datasets (Table 1). In these analyses, we defined subset similarity based solely on the overall substitution rate (i.e. we used --weights “1, 0, 0, 0”), based on our analyses of the phylogenomic dataset of birds (Figure 2), and on the results of previous phylogenomic studies that have relied on overall substitution rates to combine subsets in partitioning schemes (e.g. [29]). We fixed the proportion of partitioning schemes analysed by the relaxed clustering algorithm to 10% (i.e. --rcluster-percent 10), based on the observation that for the phylogenomic dataset of birds this cutoff represented a good balance between computational efficiency and performance. For the same reasons, we defined default settings in PartitionFinder such that subset similarity is based solely on the overall substitution rate (i.e. we used --weights “1, 0, 0, 0”), and the proportion of partitioning schemes analysed by the relaxed clustering algorithm is 10% (i.e. --rcluster-percent 10). While it is possible that these parameters are idiosyncratic to the phylogenomic bird dataset, our results below suggest that they produce broadly similar results across all of the datasets we have analysed. Furthermore, using a single set of parameters in the analyses of 10 datasets more accurately reflects the likely behaviour of the end users of these algorithms, who are unlikely to run thousands of analyses to determine the best parameters for partitioning scheme selection. Thus, using a single set of parameters represents the most useful basis for comparing the three algorithms. We provide recommendations for the use of each of these algorithms, based on the results of all of our analyses, in the Conclusions section at the end of this article.

**Figure 3:**
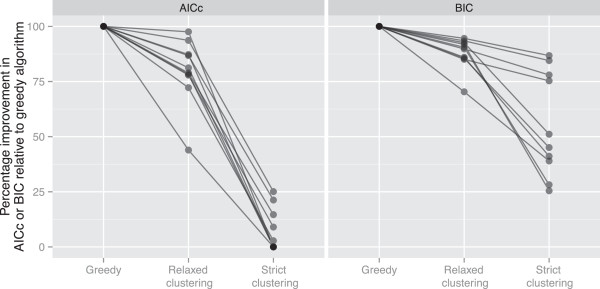
**The relaxed clustering algorithm outperforms the strict clustering algorithm across the 10 datasets shown in Table**1**.** All scores are standardised by the score increase achieved by the greedy algorithm (i.e. the score of the best partitioning scheme from the greedy algorithm minus the score of the starting scheme), so that performance can be compared across datasets. Thus, the greedy algorithm always scores 100%, and is shown only for reference. Each line connects the results from a single dataset, demonstrating that in all cases using both the AICc and the BIC, the greedy algorithm performed best, the relaxed clustering algorithm (with 10% of schemes analysed) performed second best, and the strict clustering algorithm performed the worst. All analyses use the RAxML version of PartitionFinder.

The relaxed clustering algorithm found better partitioning schemes than the strict clustering algorithm on all 10 of the datasets we examined (Figure 3, Table 2). For the relaxed clustering algorithm, the mean improvement in AICc and BIC scores across all 10 datasets was 80% and 88% of the potential improvement estimated from the greedy algorithm respectively (Figure 3, Table 2). For the strict clustering algorithm, the mean improvement in AICc and BIC scores was 7% and 55% of the potential improvement estimated from the greedy algorithm (Figure 3, Table 2).

**Table 2:** AICc and BIC scores of the best partitioning scheme found by different algorithms on each dataset

	**AICc**			**BIC**		
**Dataset**	**Greedy**	**Relaxed clustering**	**Strict clustering**	**Greedy**	**Relaxed clustering**	**Strict clustering**
	**(AICc)**	**(ΔAICc)**	**(ΔAICc)**	**(BIC)**	**(ΔBIC)**	**(ΔBIC)**
Ward_2010	103258	-34	-61	104877	-294	-606
Wainwright_2012	473537	-7	-59	477322	-73	-663
Pyron_2011	154838	-42	-173	156039	-177	-383
Li_2008	252583	-6	-242	254327	-183	-769
Leavitt_2013	424129	-216	-757	426143	-837	-3176
Kaffenberger_2011	120020	-6	-75	121452	-62	-150
Irisarri_2012	214655	-41	-187	216209	-152	-1151
Hackett_2008	1830824	-356	-1442	1837230	-964	-6362
Fong_2012	276517	-254	-1508	278400	-900	-2129
Endicott_2008	66966	-90	-479	70139	-455	-752

The greedy algorithm performed best in all cases, as expected, and the AICc/BIC score is shown for each run with that algorithm. The relaxed clustering algorithm typically performed almost as well as the greedy algorithm, and always performed better than the strict clustering algorithm. ΔAICc or ΔBIC scores are shown for the clustering algorithms, and represent the difference in AICc or BIC score from the greedy algorithm.

### The computational efficiency of the strict and relaxed clustering algorithms on 10 datasets

**Figure 4:**
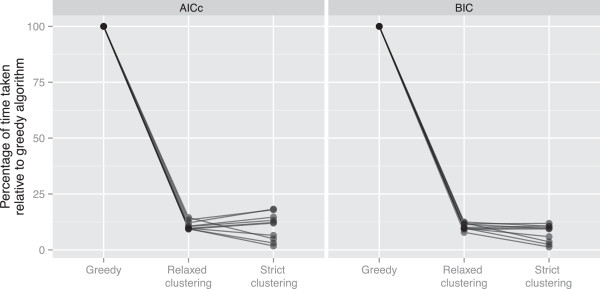
**The strict and relaxed clustering algorithms are computationally much more efficient than the greedy algorithm.** This figure shows the time taken by the relaxed and strict hierarchical clustering algorithms on the 10 datasets shown in Table 1, relative to the time taken by the greedy algorithm. All times are standardised by the time taken by the greedy algorithm, so that performance can be compared across datasets. Thus, the greedy algorithm always scores 100%, and is shown only for reference. Each line connects the results from a single dataset. The results show that the relaxed clustering algorithm (with 10% of schemes analysed) consistently takes about 10% of the time taken by the greedy algorithm, and that the strict hierarchical clustering algorithm takes between around 1% to 20% of the time taken by the greedy algorithm, depending on the dataset. All analyses use the RAxML version of PartitionFinder.

Both the relaxed clustering algorithm and the strict clustering algorithm took less computational time than the greedy algorithm, but the identity of the fastest algorithm depended on the size of the dataset (Figure 4, Table 3). The relaxed clustering algorithm was the fastest method for 6/10 datasets when using the AICc, and for 4/10 datasets when using the BIC (Figure 4, Table 3). The datasets for which the relaxed clustering algorithm was faster tended to be those with smaller numbers of data blocks. Across all datasets and information theoretic scores, the relaxed clustering algorithm finished in 11% of the time it took the greedy algorithm to finish, and the strict clustering algorithm finished in 9% of the time it took the greedy algorithm to finish. But for the two largest datasets that we analysed [43,44], the relaxed clustering algorithm finished in 9% of the time it took the greedy algorithm to finish, and the strict clustering algorithm finished in 2% of the time it took the greedy algorithm to finish (Figure 4, Table 3).

**Table 3:** Analysis times (seconds) of different algorithms on different datasets, and using different information theoretic metrics to choose partitioning schemes

	**AICc**			**BIC**		
**Dataset**	**Greedy**	**Relaxed clustering**	**Strict clustering**	**Greedy**	**Relaxed clustering**	**Strict clustering**
Ward_2010	396	42	58	587	56	58
Wainwright_2012	3305	400	603	5664	568	603
Pyron_2011	602	58	74	790	73	74
Li_2008	1246	130	165	1557	194	165
Leavitt_2013	5829	843	288	7997	973	288
Kaffenberger_2011	580	78	104	877	102	104
Irisarri_2012	935	87	112	1172	134	112
Hackett_2008	102011	9536	3140	130359	12686	3140
Fong_2012	10468	987	183	13961	1094	183
Endicott_2008	1947	189	126	2135	207	126

The two clustering algorithms are roughly an order of magnitude faster than the greedy algorithm. Analyses were conducted on a Mac Pro with 2 2.26GHz Quad-Core Intel Xeon processors and 32 GB RAM.

These differences in the speed of the strict and relaxed clustering algorithms result from two effects: search space and stopping conditions. The relaxed clustering algorithm analyses many more partitioning schemes than the strict clustering algorithm, which tends to make it slower. However, the relaxed clustering algorithm stops when the information theoretic score stops improving, whereas the strict clustering algorithm always computes the likelihood of N partitioning schemes for a dataset with N data blocks. The interplay of these two effects determines which algorithm will be quicker on any given dataset. Although the fastest algorithm depends to some extent on the number of data blocks in the optimal partitioning scheme, a general rule of thumb is that the strict clustering algorithm will be quicker on very large datasets, but will produce poorer results.

## Conclusions

Partitioning is an important part of many phylogenetic analyses, and can dramatically improve the fit of models to data for almost all datasets. This is particularly true of very large datasets, which contain more genomic regions, and thus more variation in rates and patterns of molecular evolution than smaller datasets. As the analysis of very large datasets becomes more common, methods to infer partitioning schemes need to keep pace so that we can make the best possible inferences from the datasets we have.

In this study, we compared three methods for estimating partitioning schemes: an existing greedy algorithm [2]; a strict hierarchical clustering method which extends the work of Li et al. [4]; and a relaxed hierarchical clustering method which we developed here. Our results allow us to make clear recommendations for those wishing to estimate partitioning schemes.

Preference should always be given to using the greedy algorithm in PartitionFinder over the two algorithms developed here [2]. The substantial improvements made to PartitionFinder for this study now permit the greedy algorithm to analyse datasets that include up to 200 data blocks on a desktop computer (although exact numbers will, of course, depend on the size of each data block, the number of taxa in the alignment, and the computer itself). Many datasets being collected today, however, contain hundreds or thousands of loci [23-25,44,49,50]. In these cases, it would be computationally infeasible to use the greedy algorithm to select partitioning schemes, and where possible the relaxed hierarchical clustering algorithm should be used instead.

When using the relaxed hierarchical clustering algorithm, the percentage of schemes analysed at each step of the algorithm (--rcluster-percent option in PartitionFinder) should be set as high as practically possible. Determining what is practical for a given dataset on a given computer may require some trial and error, but we suggest first running the analysis using the default setting of 10%. If this run finishes quickly, the percentage should be increased and the analysis re-run. If it runs too slowly, the analysis can be cancelled and re-started with a smaller percentage. Subsequent runs will be much faster than the initial run, because PartitionFinder saves and reloads the results of previous analyses. Determining whether a given percentage of schemes analysed will produce a partitioning scheme of a similar score to the greedy algorithm may be possible by examining the results of at least three runs of the relaxed clustering algorithm using different percentages (e.g. one with the maximum practical percentage, one with a percentage of half maximum, and one with a very small percentage). This is because as the percentage of schemes analysed is increased, the results of the relaxed clustering algorithm will asymptotically approach those of the greedy algorithm (Figure 1). Finally, if the percentage of schemes analysed is very low, then it may be prudent to perform more than one run with different sets of clustering weights.

The strict hierarchical clustering algorithm should be used only if an analysis using the relaxed hierarchical clustering algorithm is computationally infeasible. The strict hierarchical clustering algorithm is still likely to provide large improvements in the fit of the model to the data when compared to not attempting to optimise the partitioning scheme, but it may be sensible to try a number of different methods of defining subset similarity in order to ensure the best possible results (--weights option in PartitionFinder, for which the default is to define subset similarity based solely on their rates of evolution). For example, one option would be to optimise partitioning schemes under all possible combinations of setting at least one weight to 1.0, and other weights to 0.0. The best-fit partitioning scheme could then be chosen from the set of 15 estimated partitioning schemes. For simplicity, this set of 15 weights can be found in the figShare repository that accompanies this paper (http://dx.doi.org/10.6084/m9.figshare.938920).

In the future, it would be interesting to explore more complex partitioned models of molecular evolution. For example, our study considers only partitioning schemes in which each subset of sites has an independent model of molecular evolution from all other subsets. This decision was results from the practical consideration that this is the only partitioned model available in RAxML, the primary software for analysing extremely large phylogenomic datasets. However, the most recent version of other maximum-likelihood phylogenetic software, PhyML [51], allows for different subsets to share any number of parameters with any number of other subsets. This hugely increases the number of possible partitioning schemes, and in particular it allows for complex models of heterotachy to be estimated. As a result, this approach is likely to allow for partitioned models that dramatically improve on those we can currently estimate using PartitionFinder. However, searching among the space of these possible partitioned models, and estimating the optimal model for any given dataset, remains an unsolved problem.

### Availability of supporting data

The data sets supporting the results of this article are available in the figShare repository, http://dx.doi.org/10.6084/m9.figshare.938920[52]. This repository contains all of the datasets from Table 1, as well as the results of all analyses and the R script used to produce the figures in this manuscript.

All of the methods we have developed and described here of the latest version of PartitionFinder are available from: http://www.robertlanfear.com/partitionfinder.

## Competing interests

The authors declare that they have no competing interests.

## Authors’ contributions

RL and BC conceived the study. RL, BC, CM, DK, AS developed, coded, and tested the methods. RL collated and analysed the empirical datasets. RL wrote the manuscript. All authors read and approved the final manuscript.
